# Measurement of Intratumor Heterogeneity and Its Changing Pattern to Predict Response and Recurrence Risk After Neoadjuvant Chemotherapy in Breast Cancer

**DOI:** 10.3390/curroncol32020093

**Published:** 2025-02-07

**Authors:** Mingxi Zhu, Qiong Wu, Xiaochuan Geng, Huaying Xie, Yan Wang, Ziping Wu, Yanping Lin, Liheng Zhou, Shuguang Xu, Yumei Ye, Wenjin Yin, Jia Hua, Jingsong Lu, Yaohui Wang

**Affiliations:** 1 Department of Breast Surgery, Renji Hospital, School of Medicine, Shanghai Jiao Tong University, Shanghai 200127, China; aureliane@163.com (M.Z.); wqlucy3790@163.com (Q.W.); 20220@renji.com (Y.W.); wuziping@renji.com (Z.W.); linyanping@renji.com (Y.L.); zhouliheng@renji.com (L.Z.); xushuguang@renji.com (S.X.); yeyumei@renji.com (Y.Y.); yinwenjin@renji.com (W.Y.); wangyaohui@renji.com (Y.W.); 2 Department of Radiology, Renji Hospital, School of Medicine, Shanghai Jiao Tong University, Shanghai 200127, China; gengxiaochuan1949@126.com; 3 Department of Radiation Oncology, Renji Hospital, School of Medicine, Shanghai Jiao Tong University, Shanghai 200127, China; xiehuaying@renji.com

**Keywords:** breast neoplasms, magnetic resonance imaging, neoadjuvant chemotherapy, pathologic complete response, recurrence

## Abstract

The heterogeneity of breast tumors might reflect biological complexity and provide prediction clues for the sensitivity of treatment. This study aimed to construct a model based on tumor heterogeneity in magnetic resonance imaging (MRI) for predicting the pathological complete response (pCR) to neoadjuvant chemotherapy (NAC). This retrospective study involved 217 patients with biopsy-confirmed invasive breast cancer who underwent MR before and after NAC. Patients were randomly divided into the training cohort and the validation cohort at a 1:1 ratio. MR images were processed by algorithms to quantify the heterogeneity of tumors. Models incorporating heterogeneity and clinical characteristics were constructed to predict pCR. The patterns of heterogeneity variation during NAC were classified into four categories abbreviated as the heterogeneity high-keep group (H_keep group), heterogeneity low-keep group (L_keep group), heterogeneity rising group, and decrease group. The average heterogeneity in patients achieving pCR was significantly lower than in those who did not (*p* = 0.029). Lower heterogeneity was independently associated with pCR (OR, 0.401 [95%CI: 0.21, 0.76]; *p* = 0.007). The model combining heterogeneity and clinical characteristics demonstrated improved specificity (True Negative Rate 0.857 vs. 0.698) and accuracy (Accuracy 0.828 vs. 0.753) compared to the clinical model. Survival outcomes were best for the L_keep group and worst for the rising group (Log-rank *p* = 0.031). Patients with increased heterogeneity exhibited a higher risk of recurrence approaching two years post-surgery, particularly within the non-pCR population. The quantified heterogeneity of breast cancer in MRI offers a non-invasive method for predicting pCR to NAC and evaluating the implementation of precision medicine.

## 1. Introduction

Breast cancer is the most frequently diagnosed malignancy and represents the leading cause of cancer-related mortality among women. Additionally, it ranks as the second most commonly diagnosed cancer globally, accounting for 11.6% of all cases [1]. Therapeutic strategies for breast cancer are continuously evolving, demonstrating effectiveness in enhancing survival outcomes. Neoadjuvant chemotherapy (NAC) offers a strategy for surgical de-escalation [2], and the confirmation of tumor response to NAC is linked to improved disease-free survival as well as overall survival rates [3].

Somatic mutations and chromosomal imbalances may be the major prompts for phenotype diversity within the tumor, which are arbitrarily depicted as heterogeneity [4]. Genomic aberration instability is required by tumor heterogeneity, which fosters drug resistance through evolutionary selectivity under therapeutic pressure. In addition to the development of acquired resistance after treatment arising through acquired mutations, the activation of the bypass signaling pathway, and cell-lineage changes, a phenomenon known as primary resistance in preclinical tumors exists [5,6]. The efficacy of therapy could also be compromised by variations in cancer-associated fibroblasts, immune cells, and the extracellular matrix in the tumor microenvironment. Extracellular matrix stiffness may affect cell growth, disrupt cell-cell adhesions, and compromise polarity, while different vascularized stroma may inhibit drug penetration and induce hypoxia, which promotes therapeutic resistance [7]. These factors could constitute part of the material basis underlying tumor heterogeneity. A number of studies have focused on spatial heterogeneity in protein expression as a means to predict therapy sensitivity; however, these approaches inevitably rely on surgical access to tumor tissue and sample quality—limitations compounded by sequencing costs [8]. In addition to being related to treatment sensitivity, the heterogeneity of residual tumors’ pattern after NAC also affects survival outcomes [9]. There remains an urgent need to develop non-invasive technology to improve the assessment of breast cancer treated with NAC, and quantification for tumor heterogeneity based on the image of the breast cancer might mirror immense biological complexity and provide predictive and prognostic clues.

Current noninvasive clinical imaging tests to diagnose and evaluate efficacy include mammography, ultrasonography, MRI, etc. Among these modalities, MRI is considered the most accurate method for assessing tumor extent and monitoring treatment response [10]. MRI mainly utilizes the abundant hydrogen nuclei in the body in the form of bound or free water and reflects tissue density, tissue component content, and tissue perfusion by the magnitude of the grayscale intensity and may thus visualize tumor heterogeneity [11,12]. Although some previous articles have addressed the potential of MRI for detecting anatomical changes in cancer to assess response to therapy, there remains a lack of reliable noninvasive imaging modalities that are clinically accepted for quantifying the heterogeneity of breast tumors. In this report, we present an MR image processing method and computer algorithms designed to calculate tumor heterogeneity, demonstrating their capability to capture changes related to the response of breast cancer to NAC.

## 2. Materials and Methods

### 2.1. Patient Group

This retrospective study was approved by the institutional review board of Renji Hospital, School of Medicine, Shanghai Jiao Tong University (Approval number: LY2022-028-B, date: 28 October 2022). Patients were consecutively recruited from a prospective maintained database (NCT 05621564) in Renji hospital between June 2014 and September 2018. Patients were eligible if they have pathologically confirmed invasive breast cancer (T1 N1–3 or T2–4 N0–3, M0) and have received paclitaxel-and-platinum-based NAC. They should have pre-NAC MRI records with accessible images. The key exclusion criteria included metastatic or bilateral breast cancer and a history of malignancy other than breast cancer. Prospectively collected data included biological characteristics and pathology information, supplemented by record review.

### 2.2. MRI Acquisition

MR imaging was performed by using a 3-T system (Ingenia, Philips Medical Systems). Patients were examined in the prone position with the breasts naturally draped over the center of the breast coil, which was the dedicated bilateral four-channel phased-array type. Images were obtained prior to and after the injection of 0.1 mmol/kg body weight of dimeglumine gadopentetate contrast material (Magnevist; Bayer Health-care, Berlin, Germany). The parameters were as follows: TR/TE, 4.7/2.3 msec; flip angle, 10°; field of view, 320 × 320 mm^2^; matrix, 320 × 340; in-plane resolution, 1.0 × 0.9 mm; section thickness, 1 mm; and temporal resolution, 75 s.

### 2.3. MR Image Processing and Heterogeneity Definition

For a set of MR image sequences, a relatively clear presentation of breast cancer tumors image features was achieved in the dyn_eTHRIVE_5 sequence or Ax Vibrant+C sequence. The Dyn_eTHRIVE_5 sequence is a dynamically enhanced T1-weighted high-resolution isotropic volume excitation sequence, and the Ax Vibrant+C sequence is a cross-sectional bilateral 3D dynamic enhancement localization of the breast. Both clearly distinguish the tumor from the surrounding tumor bed, and the tumor interior can show different grayscale changes with contrast perfusion, serving to make heterogeneous measurements.

The processing process for each MR image from each patient was as follows: (1) find the presence or absence of a specific sequence (dyn_eTHRIVE_5 sequence or Ax Vibrant+C sequence), (2) manually identify the MR cross-section with the largest tumor area in that sequence by experienced radiologists to ensure precise and consistent annotations, (3) select the time point with the largest difference in perfusion within the tumor during the time period of dynamic enhancement, (4) take a screenshot, (5) internally circle the square area with the largest side length and save it, (6) process the intercepted square tumor image as a 12 × 12 pixel image, and then (7) measure the heterogeneity.

We used a method proposed by Brooks et al. [13] to quantify heterogeneity using grayscale intensity changing smoothness. The principle of the method is simplified as follows. Firstly, a pair of distinct object pixels labeled as *m* and *n* are picked up, with the grayscale intensity *I_m_* and *I_n_*, respectively. Then, if assuming intensity changes smoothly across the line connecting the pixel pair, any pixel between *m* and *n* named *l* could have the grayscale intensity *I*(*r_ml_*) computed as

(1) $$I\left(r_{ml}\right)=I_{m}+\frac{I_{n}-I_{m}}{r_{mn}}r_{ml}$$

*r_mn_* is the Euclidean distance between *m* and *n*. *r_ml_* is the Euclidean distance between *m* and *l*. The difference between *I*(*r_ml_*) and the actual value *I_l_* are summed up over pixels comprising the straightest line from *m* to *n* referred as *L*, as follows:

(2) $$\bar{\Delta I}=\frac{1}{L}\sum_{l\in L}\left|I\left(r_{ml}\right)-I_{l}\right|$$

**Figure 1:**
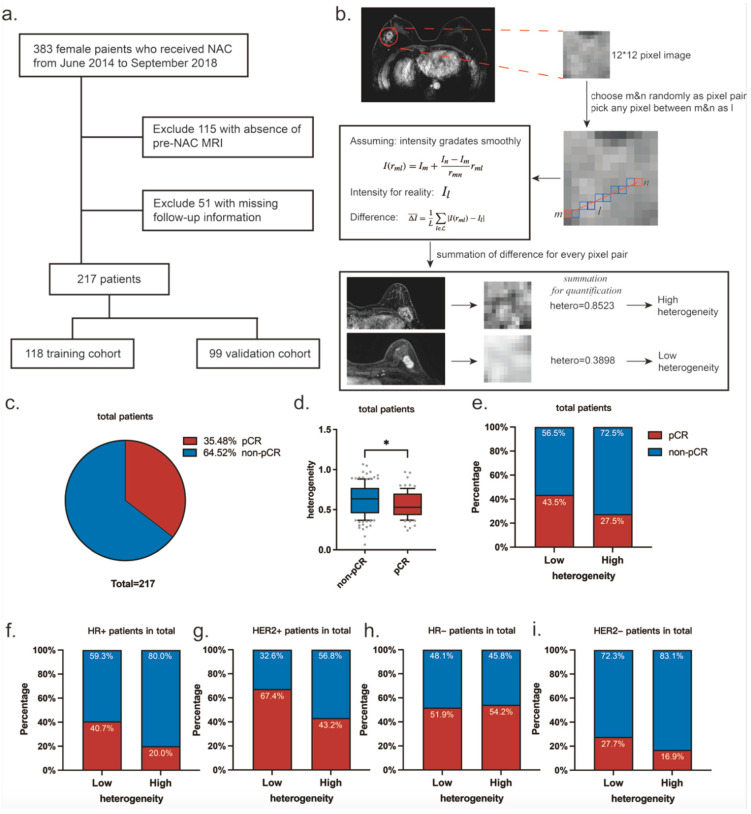
Study profile and heterogeneity feature in all. (**a**) Flowchart showing the process of selecting patients. (**b**) Work flow of the quantification of heterogeneity. (**c**) The percentages of patients who achieved pCR and those who did not in total patients. (**d**) Heterogeneity distributions in patients who achieved pCR and those who did not in total patients (* *p* = 0.029). (**e**) The percentages of patients who achieved pCR and those who did not in low-heterogeneity groups and high-heterogeneity groups. Subgroup analysis about HR status and HER2 status to demonstrate the discrepancy of heterogeneity distribution between patients who achieved pCR and those who did not (**f**–**i**).

Since the heterogeneity obtained through this algorithm is a continuous variable, the median (heterogeneity = 0.5769) was chosen as the cut-off value to classify patients into high and low heterogeneity populations. The sensitivity analyses on this threshold strengthen the conclusions (Supplementary Figure S1). The processing of the image to a uniform pixel size and the summation of the differences in gray values were implemented by Python 3.10 (Figure 1b).

The trends of heterogeneity change were classified into four categories: ‘heterogeneity persisted at a high level (abbreviated as H_keep group)’, ‘heterogeneity persisted at a low level (L_keep group)’, ‘heterogeneity rising (rising group)’, and ‘heterogeneity decreasing (decrease group)’.

### 2.4. Statistical Analysis

Cohort demographic and clinical characteristics were compared using the Chi-squared test or Fishers exact test for categorical variables. The Mann–Whitney U test was used to compare heterogeneity types with respect to non-parametric continuous variables and the t-test for parametric continuous variables. Logistic regression for correlated data was conducted to evaluate the association with pCR. The generalized estimating equation based on a multivariate logistic regression model was applied to estimate the likelihood of correct predictions as a function of clinical features and heterogeneity information upon which the prediction was based. The accuracy, sensitivity, and specificity of the model with or without heterogeneity information were calculated using a confusion matrix. Overall survival (OS), disease-free survival (DFS), and relapse-free survival (RFS) probabilities were assessed through Kaplan–Meier analysis alongside log-rank tests to ascertain the independent prognostic value. Both unadjusted and multivariable analyses of survival outcomes were performed utilizing Cox proportional hazard models, with the hazard ratio employed to quantify differences in survival rates. All statistical tests were two-sided, with *p* < 0.05 indicative of statistically significant differences. Statistical analyses were executed using R (version 3.6.0) and Stata (version 18.0).

## 3. Results

### 3.1. Patient Characteristics

Between June 2014 and September 2018, a total of 217 patients with locally advanced invasive breast cancer who underwent pre-NAC MRI were randomly assigned to either a training cohort (*n* = 118) or a validation cohort (*n* = 99) (Figure 1a). The distributions of patients in the high and low heterogeneity groups in the training and validation sets were compared in terms of patient age, menstrual status, hormone receptor (HR), human epidermal growth factor receptor 2 (HER2) status, Ki67 score, tumor T-stage, and N-stage, respectively (Table 1). The average age of participants was 51.0 years (range 23–71), with 45.2% being premenopausal. Statistical analysis revealed no significant differences in the distribution of these variables between the training cohort and validation cohort (Supplementary Table S1).

### 3.2. Variables Associated with pCR Rates

In total, 35.48% of the patients achieved pCR (Figure 1c). Our analysis revealed that the mean heterogeneity value in patients who attained pCR was significantly lower at 0.5587 compared to those who did not achieve pCR, which had a mean value of 0.6158 (*p* = 0.029, Figure 1d). Furthermore, among patients categorized into the low heterogeneity group, 43.5% achieved pCR; conversely, only 27.5% of patients in the high heterogeneity group reached this outcome (*p* = 0.016, Figure 1e).

Further analysis of our subgroups revealed that, among the HR-positive subgroup, tumoral heterogeneity significantly influenced the efficacy of neoadjuvant therapy. Patients in the low-heterogeneity group were more likely to achieve pCR compared to those in the high-heterogeneity group (40.7% vs. 20.0%, *p* = 0.004). For the HR-negative subgroup, no significant difference was observed in pCR rates between low- and high-heterogeneity groups (51.9% vs. 54.2%, *p* = 0.869). Additionally, a correlation was noted between lower tumor heterogeneity and enhanced therapeutic sensitivity within the HER2-positive subgroup; specifically, 67.4% of patients in the low-heterogeneity group achieved a pCR versus 43.2% in the high-heterogeneity group (*p* = 0.023). However, this pattern was not replicated within the HER2-negative subgroup, which exhibited pCR rates of 27.7% for low heterogeneity compared to 16.9% for high heterogeneity (*p* = 0.140) (Figure 1f–i).

**Figure 2:**
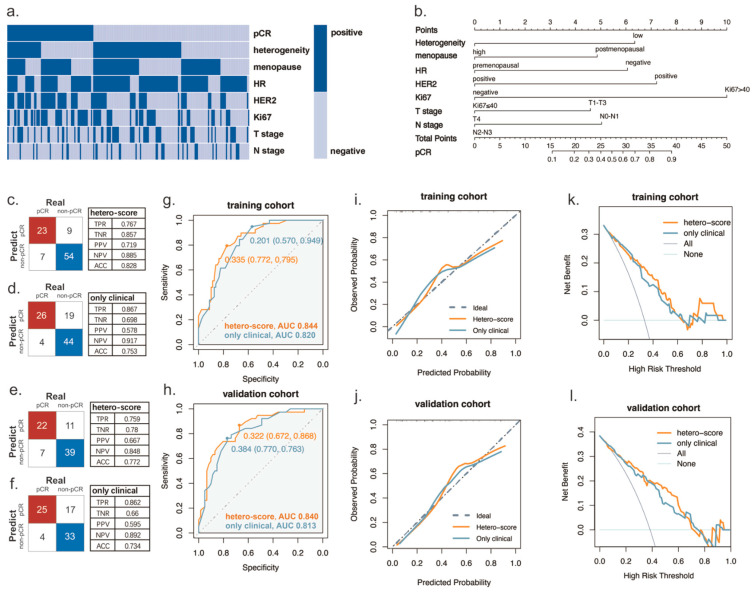
Model building and assessment for accuracy. (**a**) Heatmap with relevant indicators for predicting pCR (high-heterogeneity vs. low-heterogeneity, postmenopausal vs. premenopausal, HR positive vs. HR negative, HER2 positive vs. HER2 negative, Ki67 > 40 vs. Ki67 ≤ 40, T stage T4 vs. T1–3, and N stage N2–3 vs. N0–1). (**b**) The pathologic response prediction nomogram. Confusion matrix reflecting the predicting accuracy of the model (**c**) with clinical features and heterogeneity or (**d**) with only clinical features in the training group. The same is true in (**e**,**f**) in the validation group. The ROC for the performance of the hetero-score taking clinical features and heterogeneity into account and the criteria only considering clinical features in the (**g**) training cohort and in the (**h**) validation cohort. Calibration curves in the (**i**) training cohort (Mean absolute error = 0.053, Mean squared error = 0.0049, 0.9 Quantile of absolute error = 0.108) and (**j**) validation cohort (Mean absolute error = 0.039, Mean squared error = 0.00305, 0.9 Quantile of absolute error = 0.084). Clinical decision curves in the (**k**) training cohort and (**l**) validation cohort. TPR, true positive rate; TNR, true negative rate; PPV, positive predictive value; NPV, negative predictive value; ACC, accuracy.

Univariate logistic regression analysis of clinical characteristics in the entire group indicated that the HR status values (odds ratio [OR], 0.383 [95% confidence interval (CI): 0.202, 0.728]; *p* = 0.003), HER2 status (OR, 4.284 [95%CI: 2.374–7.739]; *p* < 0.001), and Ki67 score (OR, 2.707 [95%CI: 1.524, 4.809]; *p* = 0.001) were all significantly associated with achieving pCR (Table 2, Figure 2a). Patients with low heterogeneity were more likely to attain pCR (OR, 0.493 [95%CI: 0.28, 0.87]; *p* = 0.014).

Multivariable analysis revealed that lower heterogeneity remained independently associated with an increased likelihood of achieving pCR (OR, 0.401 [95%CI: 0.21, 0.76]; *p* = 0.007). Furthermore, the HR status (OR, 0.387 [95%CI: 0.19, 0.81]; *p* = 0.012), HER2 status (OR, 5.298 [95%CI: 2.65–10.60]; *p* < 0.001), and Ki67 score (OR, 2.927 [95%CI: 1.52–5.63]; *p* = 0.001) also served as independent predictors for pCR simultaneously (Table 2). Univariable subgroup analysis revealed that patients with low heterogeneity were more likely to achieve pCR within the HR+ subgroup (*p* = 0.004), the HER2+ subgroup (*p* = 0.024), the Ki67 > 40% subgroup (*p* = 0.008), the T1–3 stage subgroup (*p* = 0.039), and the N0–1 stage subgroup (*p* = 0.017) (Figure 1f–i). No significant interaction was observed between heterogeneity and each clinical characteristic (Supplementary Table S2).

### 3.3. Prediction pCR by Using a Multivariate Model

We developed a prediction model for pCR after NAC based on logistic regression analysis. Our study underscores the potential significance of heterogeneity in prediction by constructing two models: one that incorporates heterogeneity and another that does not. A confusion matrix was generated based on the model containing heterogeneity versus that without heterogeneity in the training and validation cohorts, respectively. The results demonstrated that in the training cohort, the model combining heterogeneity with clinical features exhibited superior specificity (true negative rate [TNR] 0.857 vs. 0.698) as well as enhanced accuracy (ACC 0.828 vs. 0.753). Similarly, within the validation set, the model integrating heterogeneity alongside clinical features outperformed the sole clinical features model (TNR 0.780 vs. 0.660; ACC 0.772 vs. 0.734) as illustrated in Figure 2c–f. In comparison to the non-heterogeneous model, our predictive framework incorporating both clinical characteristics and measures of heterogeneity demonstrated improved efficacy in predicting pCR, achieving an area under the receiver operating characteristic curve (AUC) of approximately 0.844 in the training cohort versus an AUC of only 0.820 for its counterpart utilizing solely clinical features. In the validation cohort, there was also a notable enhancement in AUC when employing a heterogeneous approach compared to using only clinical factors (AUC: 0.837 vs. 0.813) (Figure 2g,h).

We also evaluated the performance of the predictive models using calibration curves (Figure 2i,j), which indicated that the heterogeneous joint clinical feature model demonstrated higher accuracy. The clinical decision curve analysis revealed that the model incorporating heterogeneity provided greater benefits compared to the non-heterogeneous model across a broad range of threshold probabilities (Figure 2k,l). In addition, based on the training set, a nomogram was developed for predicting the pathologic response by integrating heterogeneity with other key clinical features (Figure 2b).

### 3.4. Survival Analysis

**Figure 3:**
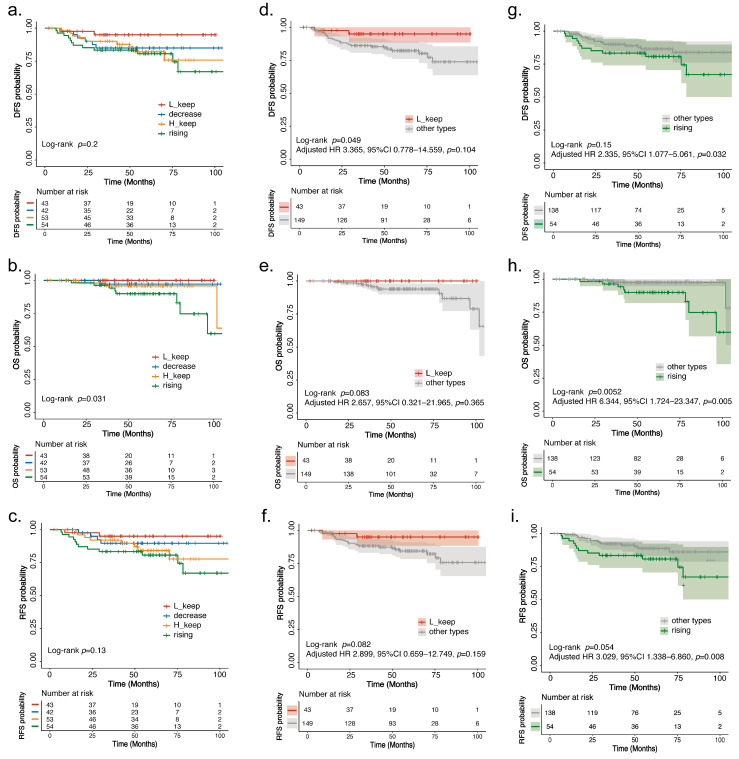
Kaplan–Meier survival curve analyses of breast cancer patients according to heterogeneity changing trends. After classifying the change trends of heterogeneity into four categories: “H_keep”, “L_keep”, “rising”, and “decrease”, the survival curves regarding (**a**) DFS, (**b**) OS, and (**c**) RFS were plotted respectively. After classifying the change trends of heterogeneity into two categories, namely “L_keep” and “other types”, the survival curves regarding (**d**) DFS, (**e**) OS, and (**f**) RFS were plotted respectively. After classifying the change trends of heterogeneity into two categories, namely "rising" and "other types", the survival curves regarding (**g**) DFS, (**h**) OS, and (**i**) RFS were plotted respectively. H_keep, Heterogeneity continues to be at high; L_keep, Heterogeneity continues to be at low; rising, rising heterogeneity after NAC; decrease, decreased heterogeneity after NAC.

Univariate survival analysis indicated that patients exhibiting different patterns of heterogeneity change demonstrated significantly varied OS rates (Figure 3). The L_keep group had the most favorable survival outcomes, whereas the rising group exhibited the poorest survival performance (Log-rank *p* = 0.031; Hazard ratio [HR] = 8.85 [95%CI 0.494, 0.873]) (Figure 3b). Similar trends in survival were noted for DFS and RFS, although these did not reach statistical significance (DFS *p* = 0.2; RFS *p* = 0.13). Notably, the OS rate of the rising group showed a statistically significant difference when compared to other groups (Log-rank *p* = 0.005; HR = 7.82 [95%CI 0.494–0.873]) (Figure 3h).

The rising trend of heterogeneity observed during neoadjuvant chemotherapy serves as an independent poor prognostic indicator after adjusting for clinical factors such as HR, HER2 status, Ki67 score, T stage, N stage, and pCR (Figure 3g–i). Compared with the other three categories, the rising heterogeneity group showed significantly poorer OS (HR, 6.344 [95%CI: 1.724, 23.347]; *p* = 0.005), DFS (HR, 2.335 [95%CI: 1.077, 5.061]; *p* = 0.032) and RFS (HR, 3.029 [95%CI: 1.338, 6.860]; *p* = 0.008). Subgroup analysis revealed that tumors characterized by rising heterogeneity were significantly associated with shorter DFS when compared to other changing trends—particularly in premenopausal patients (HR, 7.558; *p* = 0.004), HR-positive cases (HR, 3.461; *p* = 0.034), HER2-negative tumors (HR, 4.259; *p* = 0.025), those with Ki67 ≤40% (HR, 5.545; *p* = 0.015), and those within the T4 subgroup (HR, 3.684; *p* = 0.022) (Supplementary Figure S2). Similar patterns were also observed in RFS analyses across these subgroups.

**Figure 4:**
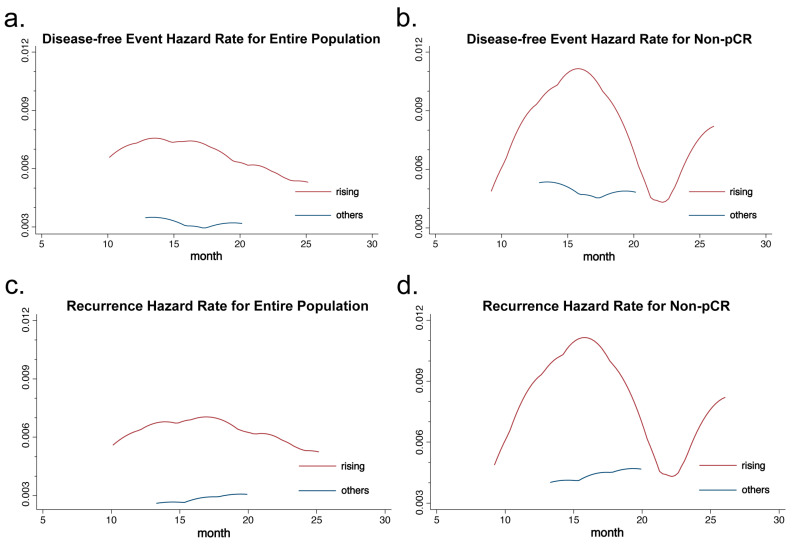
Disease-free event hazard rates for (**a**) the entire population and (**b**) patients who did not reach pCR. Recurrence hazard rates for (**c**) the entire population and (**d**) patients who did not reach pCR.

Recurrence hazard curves comparing the heterogeneity rising group and others showed that the rising group experienced a peak in recurrence at approximately the 15th month. Meanwhile, other heterogeneity trend groups presented a stable lower level of recurrence risk without any discernible peak (Figure 4a,c). Similar findings were noted in patients within the non-pCR subgroup; however, the magnitude of the difference between these two groups was even more pronounced (Figure 4b,d).

## 4. Discussion

Tumor heterogeneity and its fluctuations during treatment may provide crucial insights into the underlying mechanisms of treatment sensitivity, prognosis, and the spatial and temporal recurrence and metastasis of tumors. This study pioneered a non-invasive method for quantifying tumor heterogeneity measured through MRI before and after NAC and established categories for changes in trend in heterogeneity. To our knowledge, it was first observed that the trends in heterogeneity changes could have prognostic implications; specifically, patients whose heterogeneity increases throughout treatment may experience poorer outcomes. The time to recurrence showed significant differences between patients exhibiting rising heterogeneity compared to those with alternative changing patterns, particularly within the non-pCR subgroup. These initial findings offer novel insights into non-invasive prognostication regarding responses to NAC, prediction of metastatic recurrence timing, patient risk assessment, and efficient identification of candidates who may benefit from intensified postoperative adjuvant therapy. This is especially pertinent for non-pCR patients requiring escalation of treatment following adjuvant therapy.

Our study indicated that breast cancers exhibiting high levels of pre-NAC heterogeneity on MRI would face challenges in achieving pCR. The heterogeneity depicted in MRI seems to scratch the surface phenomenon of tumor heterogeneity; however, due to MRI, it may reflect tumor density, component, and perfusion and the heterogeneity of MRI may harbor microenvironmental inputs, variations in gene expression, metabolic richness, etc., thus forming a precise microcosm of tumor heterogeneity [14]. The diversity of cellular components and subtypes contributes significantly to the heterogeneity of the tumor microenvironment. For instance, cancer-associated fibroblasts exhibit various cellular subtypes; among them, CD10+GPR77+ cancer-associated fibroblasts continuously secrete IL-6 and IL-8 to maintain the differentiation potential of tumor stem cells, thereby inducing the onset of drug resistance [15]. In addition, the structural and microenvironmental alterations induced by tumor progression lead to adaptive shifts in tumor cell metabolism, resulting in metabolic heterogeneity attributable to metabolic reprogramming. The significant metabolic heterogeneity within the tumor microenvironment contributes to the variability in tumor tissue response to therapy [16]. In contrast, targeting and inhibiting tumor plasticity could yield therapeutic outcomes exceeding initial expectations. For example, combination treatment with trastuzumab and ARRY380 for HER2-positive breast cancer would lead to a reduction in cancer plasticity and a superior antiproliferative effect [17]. Also, some studies have indicated that the normalization of blood vasculature could improve drug delivery as well as reduce microenvironmental heterogeneity, inhibiting selection for more aggressive subpopulations [18]. Thus, far-reaching evidence aligns with our observation that tumors characterized by high levels of MRI-based heterogeneity are more likely to exhibit treatment resistance.

This study demonstrated that increased MRI heterogeneity in breast cancer during NAC may predict poorer survival and vice versa those who attained a lower heterogeneity through therapy may have a better prognosis. These results suggested the temporal heterogeneity of breast tumors while undergoing treatment. The trunk of the tumor evolutionary tree signifies early somatic events that drive tumor growth or maintenance, while the branched separation of subclones represents heterogeneous aberrations [19]. Fluctuation in heterogeneity observed before and after NAC could reflect the remodeling of tumor tissue by treatment as well as the evolution of the tumor. The clinical implication derived from our findings indicates that pre-therapy MRI heterogeneity should not be considered in isolation; rather, it must be compared with post-therapy heterogeneity to elucidate the selection pressures on tumor evolution imposed by different treatment regimens.

Achieving pCR at surgery following NAC is associated with improved survival outcomes, while those with residual disease are recommended to undergo further adjuvant intensive therapy in accordance with clinical guidelines. It has become standard practice that patients demonstrating evidence of residual triple-negative breast cancer post-NAC should accept adjuvant capecitabine for a duration of 6–8 cycles [20]. Moreover, for HER2-positive early breast cancer patients who exhibit residual invasive disease after NAC, the use of adjuvant trastuzumab emtansine has shown significant potential in reducing recurrence risk [21]. One of the most compelling insights from this study was the identification of patients with increased heterogeneity after NAC who exhibited a markedly elevated risk of recurrence approaching two years post-surgery, particularly within the non-pCR population. The noted peak in recurrence was significant at one year following surgery among those classified as having rising heterogeneity. Conversely, other heterogeneity-changing groups displayed consistently low risks of recurrence without any significant recurrence peaks. Tumor evolution is dynamic and could be modified by treatment exposures. Tumor heterogeneity partly manifests the extent of cancer cell plasticity, empowering an ability to drastically alter biological properties and leading to new phenotypes that contribute to tumor drug resistance and metastasis [16,22]. A residual disease characterized by rising heterogeneity may display distinctive patterns indicative of reawakening from tumor dormancy. This finding introduces valuable screening criteria for follow-up strategies aimed at intensifying adjuvant treatment within clinical settings—particularly benefiting those patients identified as being at heightened risk for metastatic recurrence or facing high odds of clinical relapse.

This study has some limitations. Initially, the number of patients included in our study was insufficient. A larger sample size would likely yield improved results regarding survival rates and enhance the accuracy of prediction models and nomogram construction. Moreover, there is a lack of external validation for our findings. In addition, a certain number of patients miss MR images after NAC, leading to an inadequate statistical analysis of heterogeneity-changing trends, which is inherent to the retrospective nature of our study. Despite this, the current results demonstrate a clear and significant relationship between the trend of heterogeneity changes and survival outcomes. Expanding the sample size and addressing data completeness in future studies will be crucial for further validating these findings. Lastly, considerable potential for enhancement in image processing algorithms remains; we aspire to integrate artificial intelligence for rapid image processing to minimize errors associated with the manual delineation of areas of interest, thereby making calculations more concise, efficient, and accessible.

## 5. Conclusions

This study has established an algorithm for quantifying the heterogeneity of breast cancer in MRI. The alterations in heterogeneity patterns during neoadjuvant therapy hold significant prognostic implications, highlighting the informative potential of temporal heterogeneity in breast cancer to inform future intensive treatment strategies. Additionally, we constructed a pCR prediction model involving heterogeneity and clinical features that achieved excellent performance. A prospective study is warranted to further investigate the association between trends in heterogeneity changes during neoadjuvant treatment and subsequent recurrence risk.

## Figures and Tables

**Table 1:** Clinical and histopathologic characteristics of patients grouped by heterogeneity.

Variables	Training Cohort			Validation Cohort			Total
	High Heterogeneity = 61	Low Heterogeneity = 57	*p*	High Heterogeneity = 48	Low Heterogeneity = 51	*p*	
Median age, years (range)			0.87			0.705	
	51.2 (28–69)	50.9 (23–71)		51.3 (30–69)	50.5 (26–68)		51.0 (23–71)
Menopausal status, n (%)			0.681			0.499	
Premenopausal	28 (45.9%)	24 (42.1%)		24 (50.0%)	22 (43.1%)		98 (45.2%)
Postmenopausal	33 (54.1%)	33 (57.9%)		24 (50.0%)	29 (56.9%)		119 (54.8%)
Hormone receptor status, n (%)			0.527			0.943	
Positive	48 (78.7%)	42 (73.7%)		37 (77.1%)	39 (76.5%)		166 (76.5%)
Negative	13 (21.3%)	15 (26.3%)		11 (22.9%)	12 (23.5%)		51 (23.5%)
HER2 status, n (%)			0.501			0.395	
Positive	23 (37.7%)	25 (43.9%)		21 (43.8%)	18 (35.3%)		87 (40.1%)
Negative	38 (62.3%)	32 (56.1%)		27 (56.2%)	33 (64.7%)		130 (59.9%)
Ki67 score, n (%)			0.417			0.98	
Ki67 ≤ 40	34 (55.7%)	36 (63.2%)		30 (62.5%)	32 (62.7%)		132 (60.8%)
Ki67 > 40	27 (44.3%)	21 (36.8%)		18 (37.5%)	19 (37.3%)		85 (39.2%)
T stage, n (%)			0.199			0.844	
T1–T3	37 (60.7%)	41 (71.9%)		33 (68.8%)	36 (70.6%)		147 (67.7%)
T4	24 (39.3%)	16 (28.1%)		15 (31.2%)	15 (29.4%)		70 (32.3%)
N stage, n (%)			0.357			0.73	
N0–N1	51 (83.6%)	51 (89.5%)		39 (81.3%)	40 (78.4%)		181 (83.4%)
N2–N3	10 (16.4%)	6 (10.5%)		9 (18.7%)	11 (21.6%)		36 (16.6%)

**Table 2:** Univariate and multivariate analysis for the predictive clinical features of pCR in all patients.

Variables	Comparison	Univariate Analysis			Multivariate Analysis		
		OR	95%CI	*p* Value	OR	95%CI	*p* Value
Menopausal status	Post vs. Pre	1.065	0.609–1.863	0.825	0.711	0.357–1.415	0.331
HR	Positive vs. Negative	0.383	0.202–0.728	0.003	0.387	0.185–0.810	0.012
HER2	Positive vs. Negative	4.284	2.374–7.739	<0.001	5.298	2.649–10.595	<0.001
Ki67	>40 vs. ≤40	2.707	1.524–4.809	0.001	2.927	1.521–5.630	0.001
T stage	T4 vs. T1–T3	0.632	0.341–1.169	0.143	0.45	0.264–1.591	0.344
N stage	N2–N3 vs. N0–N1	0.554	0.246–1.248	0.154	0.648	0.330–2.188	0.737
Heterogeneity	High vs. Low	0.493	0.280–0.869	0.014	0.401	0.208–0.775	0.007

## Data Availability

Data available on request due to restrictions (e.g., privacy, legal, or ethical reasons).

## Supplementary Materials

The following supporting information can be downloaded at https://www.mdpi.com/article/10.3390/curroncol32020093/s1: Figure S1: Sensitivity analyses for cut-off points; Figure S2: Subgroup Analysis of DFS in heterogeneity changing pattern; Table S1: Clinical and histopathologic characteristics of patients in training cohort and validation cohort; Table S2: Subgroup analysis for pCR by heterogeneity.

## Abbreviations

The following abbreviations are used in this manuscript:

ACC	Accuracy
AUC	Area under the curve
CI	Confidence interval
DFS	Disease-free survival
HER2	Human epidermal growth factor receptor 2
HR	Hormone receptor
NAC	Neoadjuvant chemotherapy
NPV	Negative predictive value
OR	Odds ratio
OS	Overall survival
pCR	Pathologic complete response
PPV	Positive predictive value
RFS	Relapse-free survival
TNR	True negative rate
TPR	True positive rate
