# Supplementary figures and images for: Measurement of Intratumor Heterogeneity and Its Changing Pattern to Predict Response and Recurrence Risk After Neoadjuvant Chemotherapy in Breast Cancer
Source: Curr Oncol. 2025 Feb 7;32(2):93. doi: 10.3390/curroncol32020093 (PMC11853886; doi:10.3390/curroncol32020093)

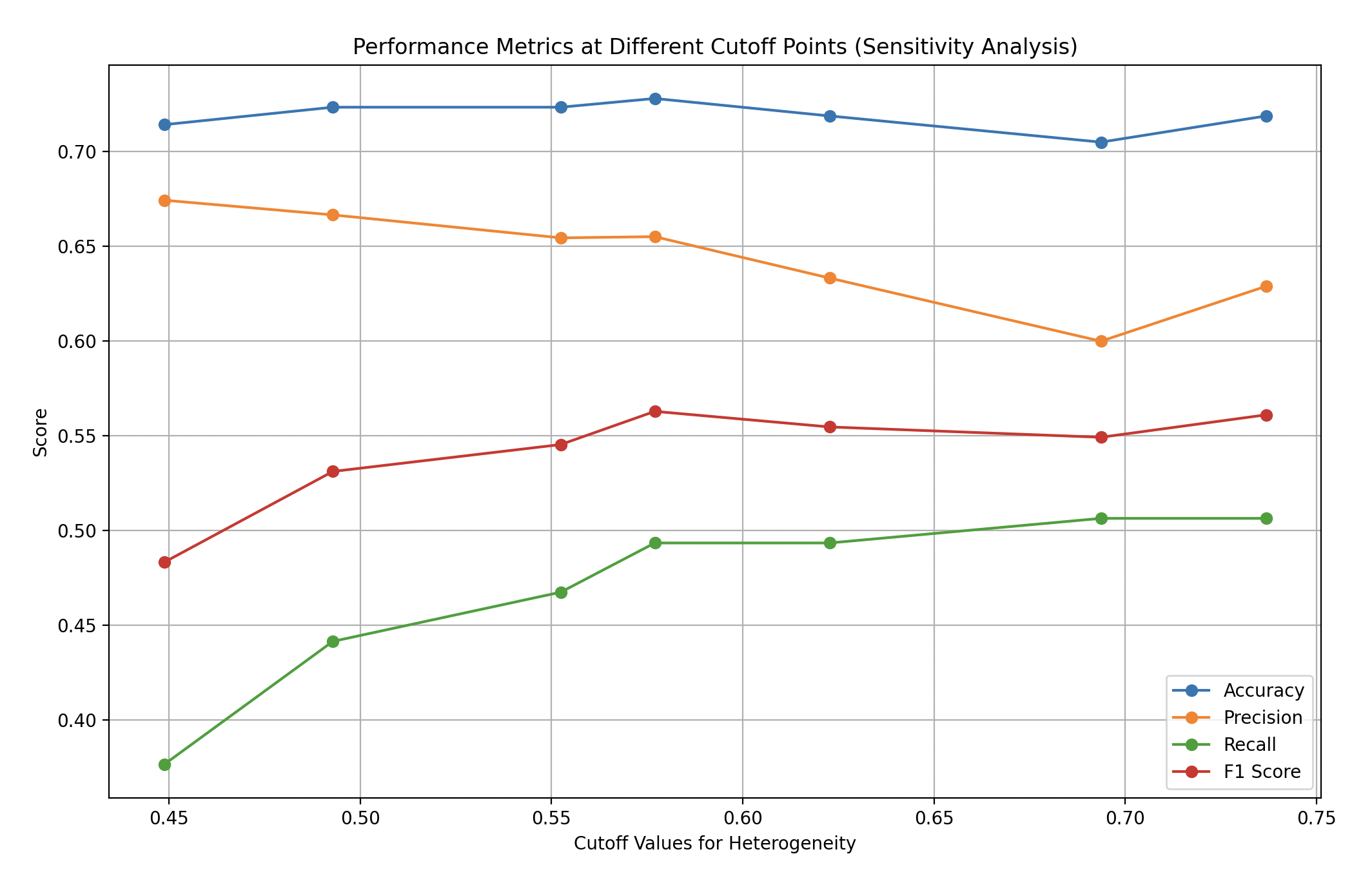

Supplement: Supplementary file 1 [file curroncol-32-00093-s001.zip › Supplementary FigureS1.png]

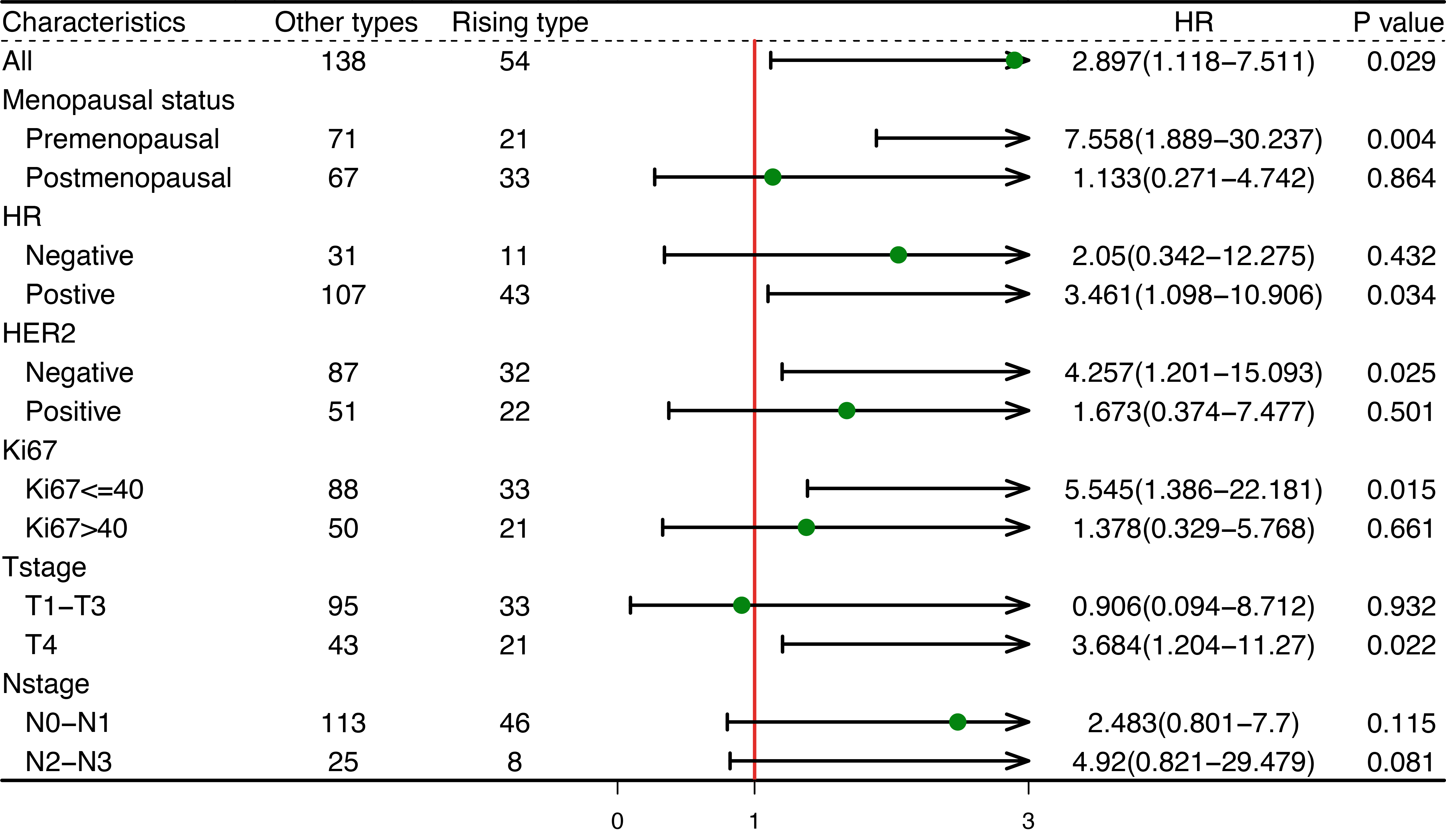

Supplement: Supplementary file 1 [file curroncol-32-00093-s001.zip › Supplementary FigureS2_Subgroup Analysis of DFS.tif]
